# Supplementary material for: Nb RD21 protease controls receptor kinase homeostasis in Nicotiana benthamiana
Source: New Phytol. 2026 Jul 12;251(5):2741–52. doi: 10.1111/nph.71407 (PMC13443273; doi:10.1111/nph.71407)
Supplement: Supplementary file 1 [file NPH-251-2741-s001.pdf]

New Phytologist Supporting Information

Article title: ***Nb*****RD21 protease controls receptor kinase homeostasis in *Nicotiana benthamiana***

Authors: Alice Godson[1], Lauren Eddie[1], Mariana Schuster[1], Kaijie Zheng[1], Reka Toth[2], Yuge Li[1,3], Tianrun Li[1], Jie Huang[1], Farnusch Kaschani[4], Philippe V. Jutras[1], Jiorgos Kourelis[1], Markus Kaiser[5], Tolga Bozkurt[6], Renier A. L. van der Hoorn[1,]*

Article acceptance date: 10 June 2026

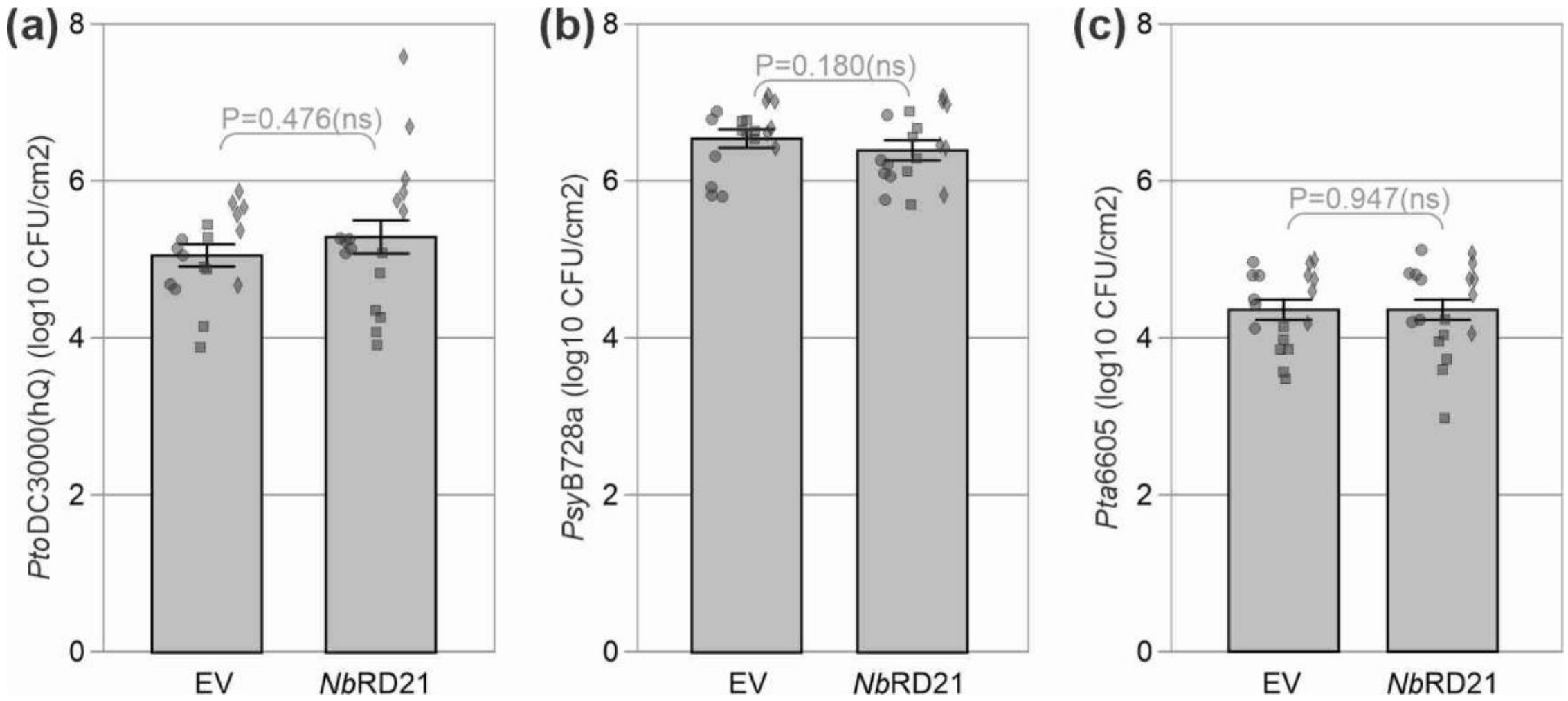

Fig. S1 No differential bacterial growth in *rd21* mutants for virulent *P. syringae* strains.

*Nb*RD21 and the EV control were transiently co-expressed by agroinfiltration in 4-week-old *N. benthamiana* with silencing inhibitor P19. Two days later, the same leaves were infiltrated with 10^6 CFU/mL *P. syringae* pathovars DC3000(*ΔhopQ1-1*) **(a)**, pv. *syringae* B728a **(b)** and pv. *tabaci* 6605 **(c)**. Bacterial growth in colony-forming units (CFU) was measured at 3 days after *Pto*DC3000 inoculation. Bars represent SE of n=6 replicates of three experiments. P-values were calculated by two-way ANOVA followed by post-hoc comparison using the Dunnett test. Ns, non-sigificant.

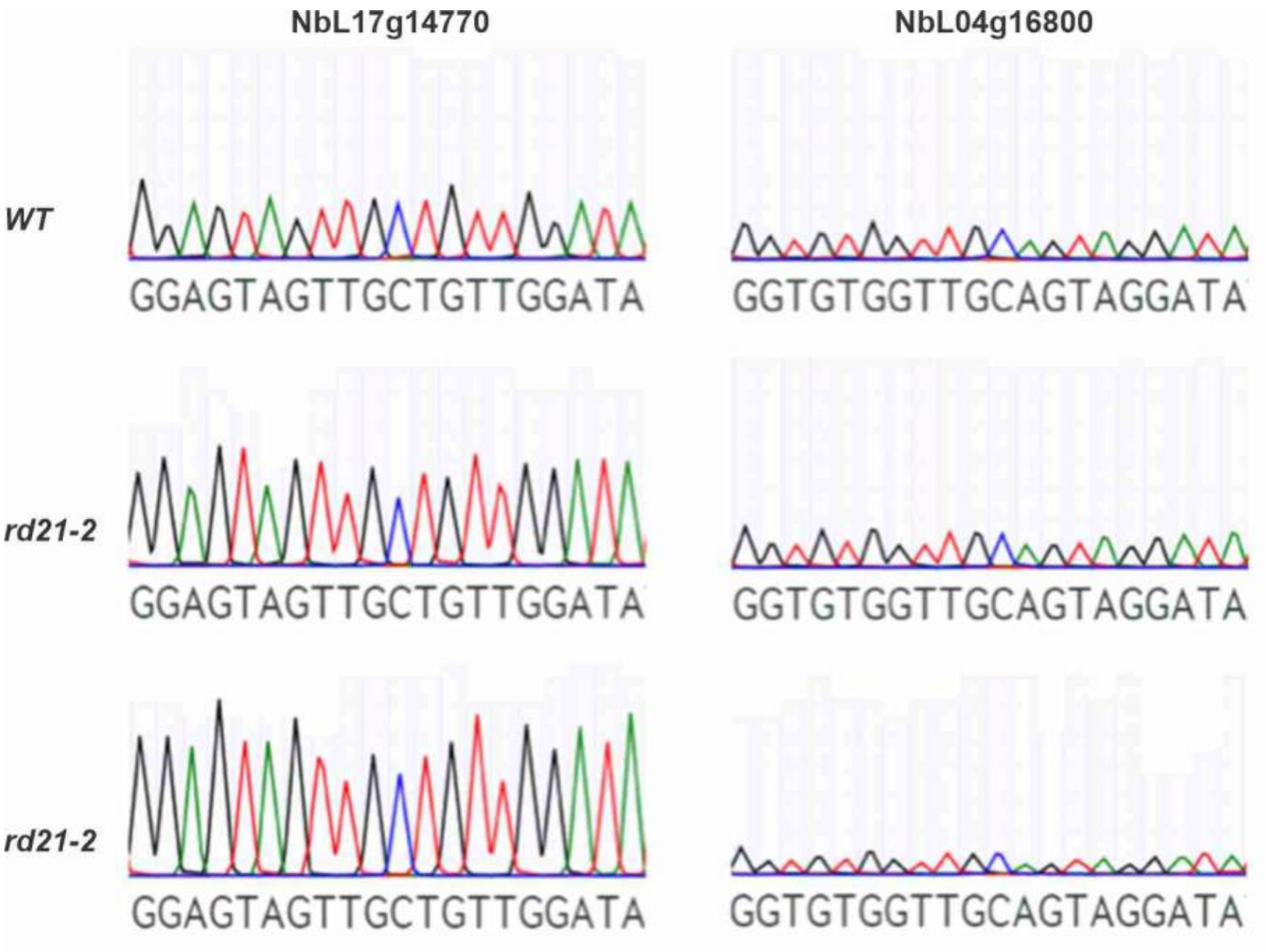

Fig. S2 Unaltered off-targets of used sgRNAs.

Cas-OFFinder identified sequences in the *N. benthamiana* genome with ≤3 mismatches with either sgRNA targeting NbL08g19980, returning RD21 homologs NbL17g14770 and NbL04g16800. Genes from wildtype (WT) and both *rd21* lines were sequenced and analysed using Benchling.com.

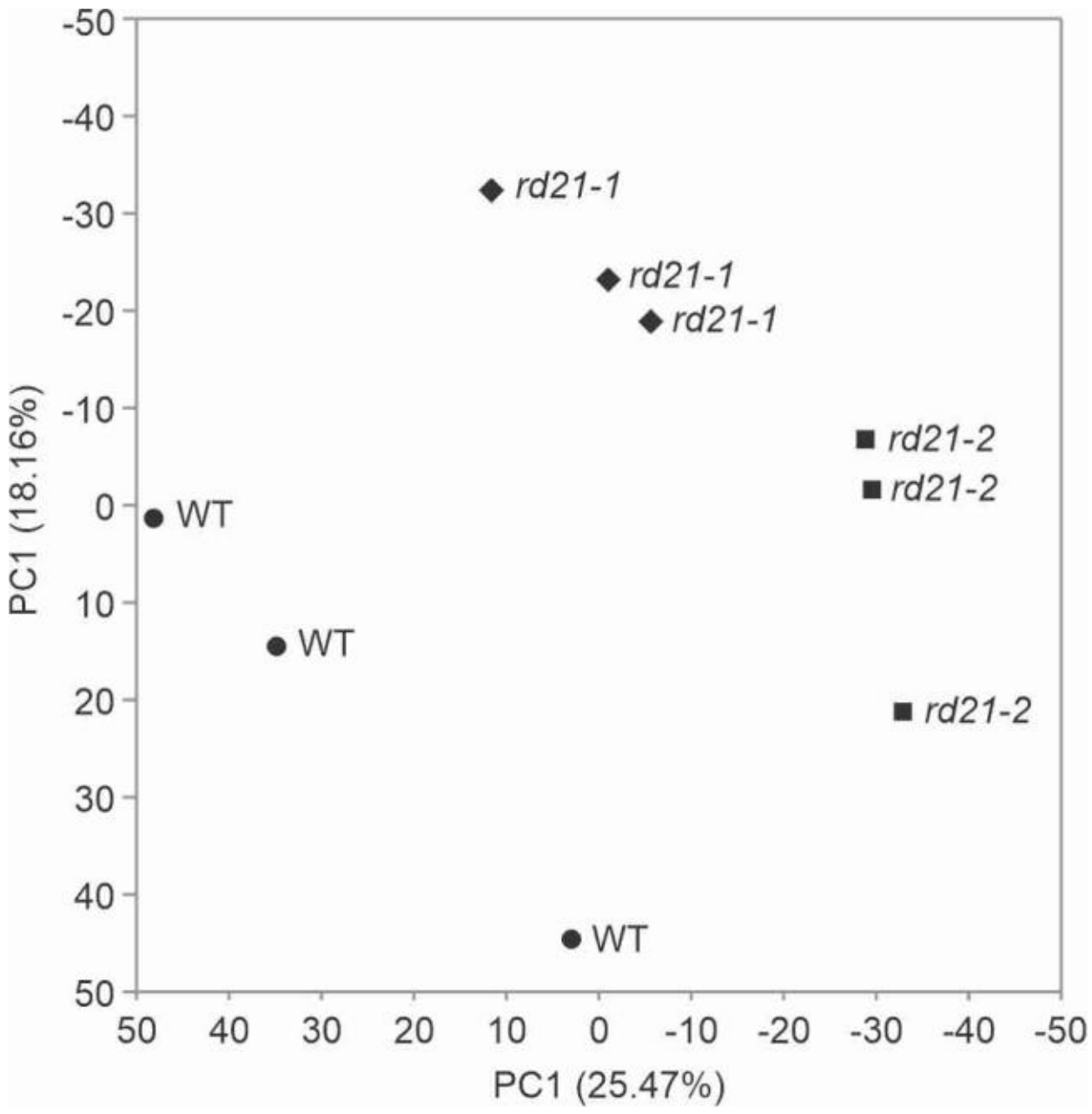

Fig. S3 PCA analysis of the shot gun proteomics on the nine samples.

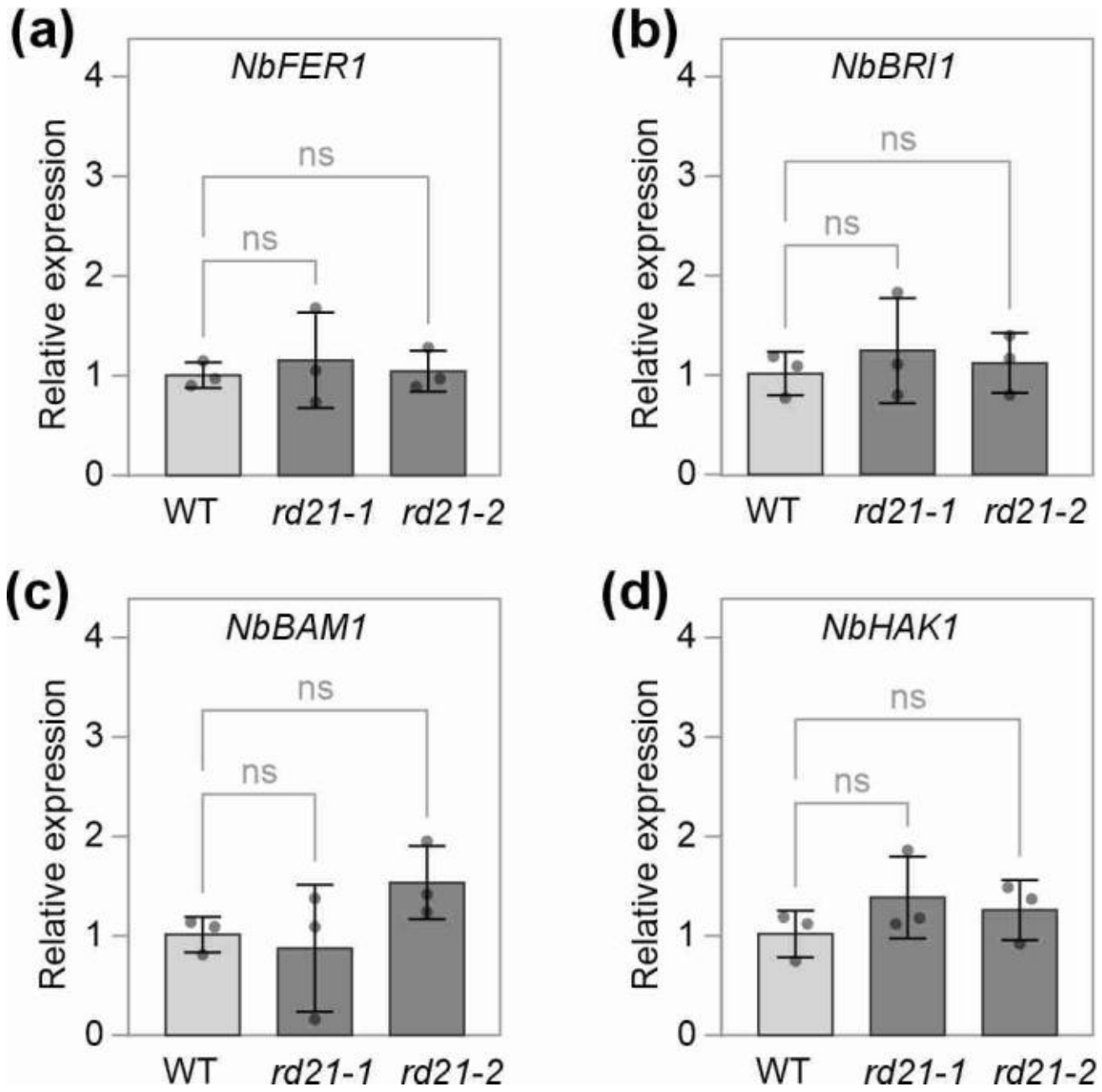

Fig. S4 Transcript levels of RLK-encoding genes.

Transcript levels were determined in n=3 plants of WT, *rd21-1* and *rd21-2* genotypes using gene specific primers, and *eIF4A* as a control, and normalized to WT plants. Error bars represent standard deviation of n=3 replicates. Statistical significance was evaluated using ANOVA plus Dunnett's multiple comparison. ns, not significant.

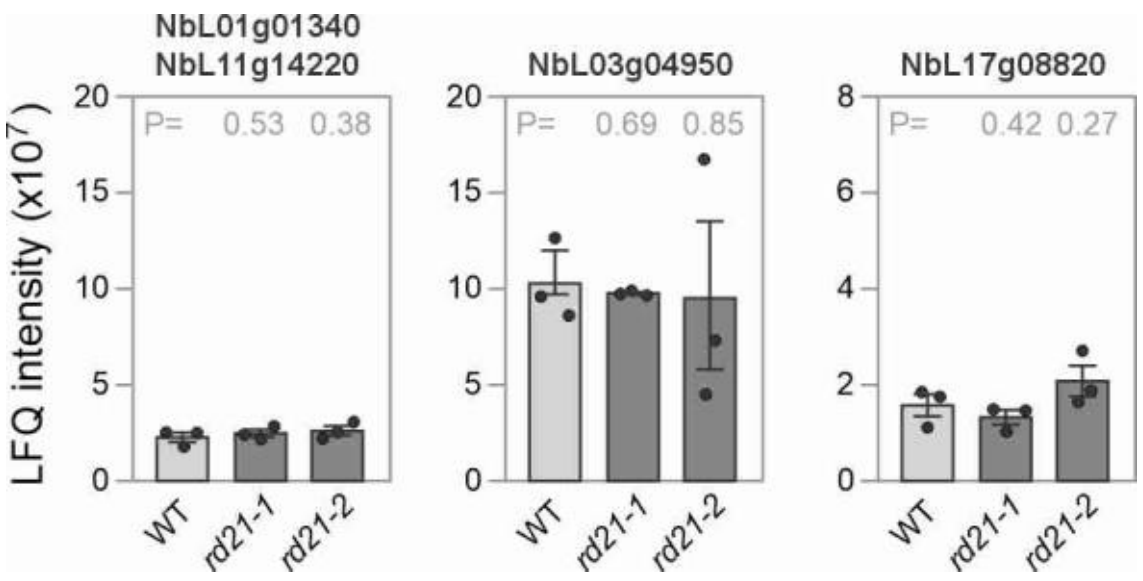

Fig. S5 No increased accumulation of three RLKs in *rd21* plants

Shown are raw LFQ intensities in wildtype (WT) and both knockout (*rd21-1* and *rd21-2*) plants. Error bars represent SE of n=3 samples. P-values calculated with Student's *t*-test compared to WT were all non-significant.

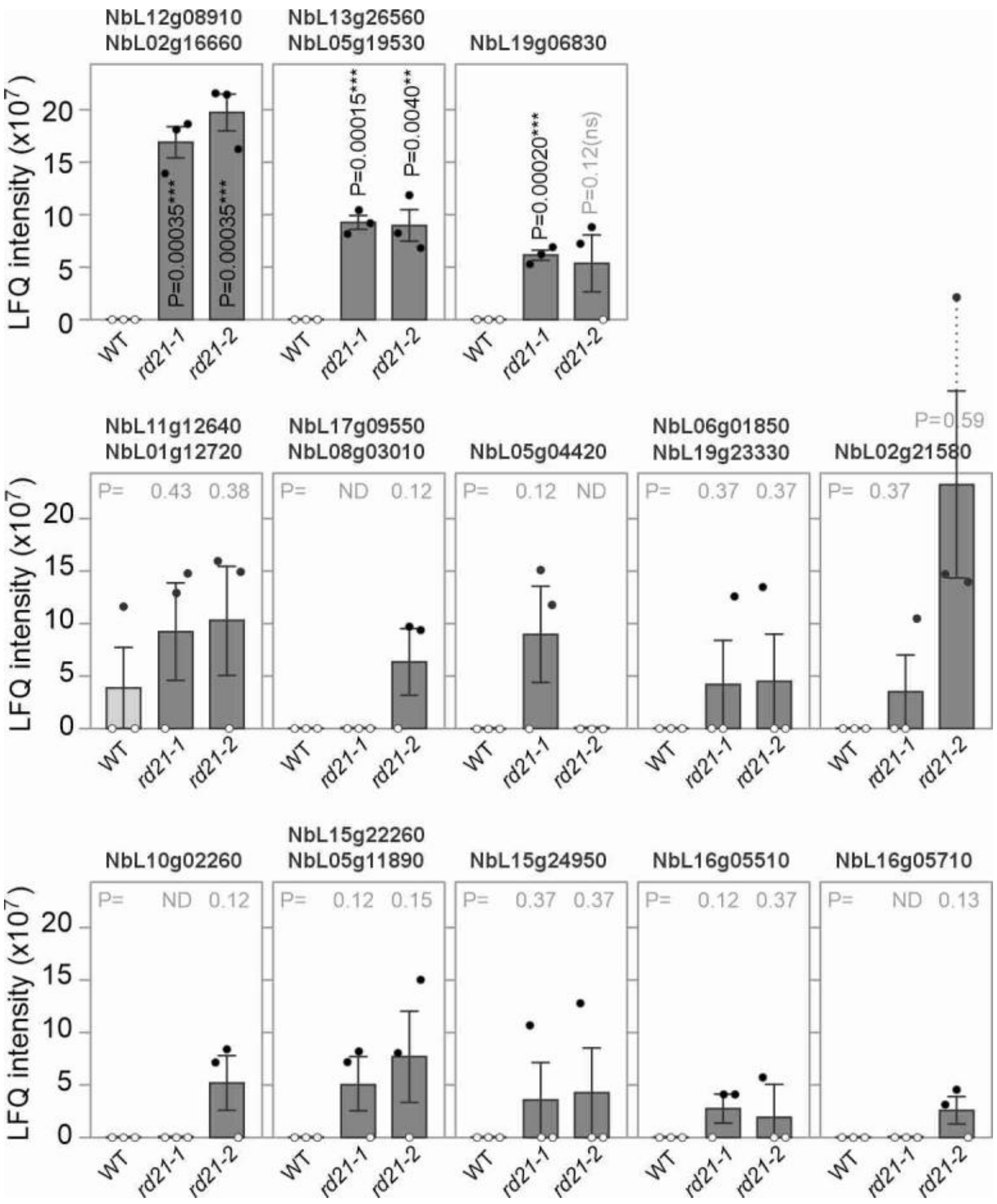

Fig. S6 13 additional RLKs seem to accumulate more in *rd21* mutants.

Bar charts showing raw LFQ intensities in wildtype (WT) and both knockout (*rd21-1* and *rd21-2*) plants. Open dots indicate the absence of a detectable MS signal. Error bars represent SE of n=3 samples. Student's *t*-test compared to WT values: **, p<0.01; ***, p<0.001. ND, not determined.

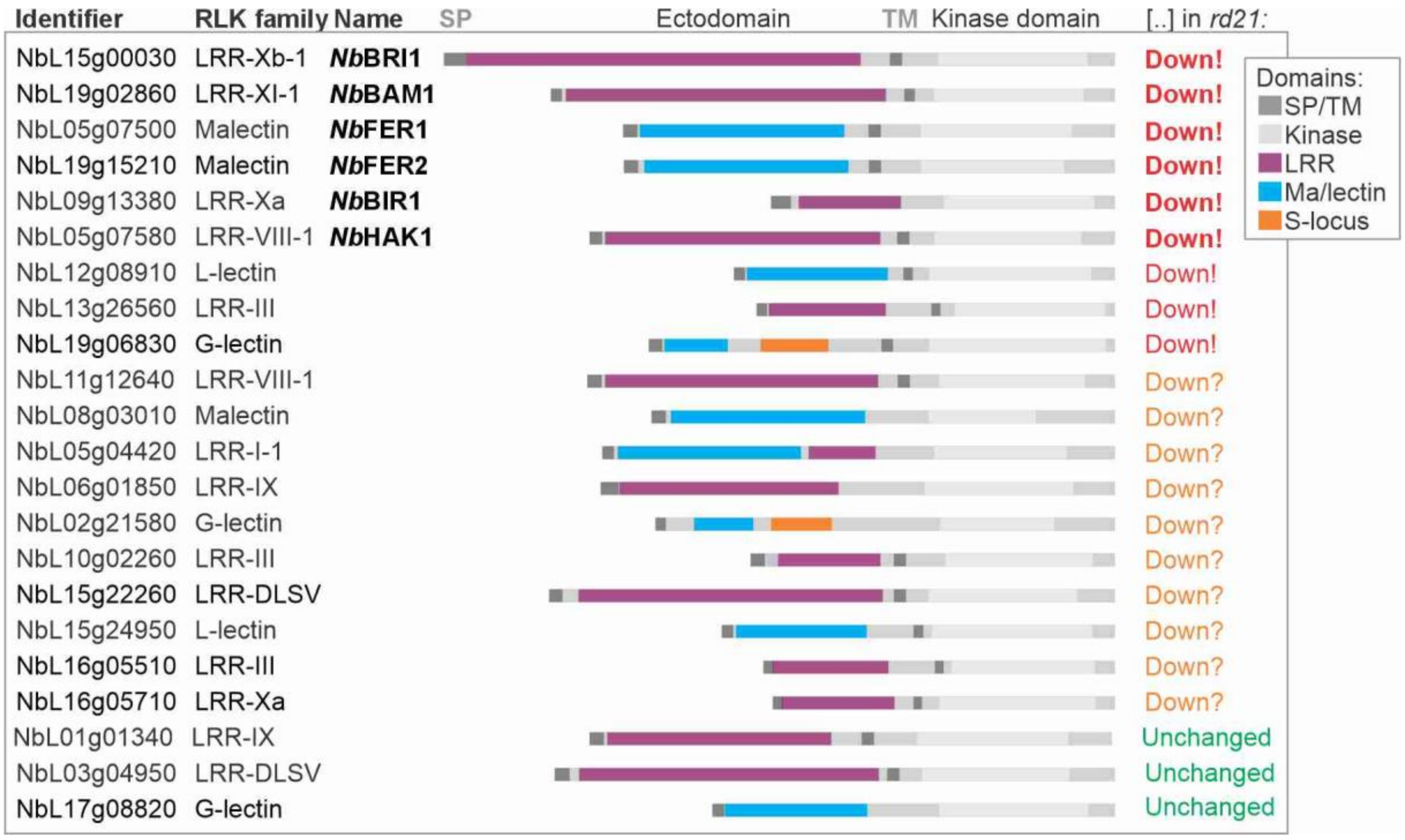

Fig. S7 Domain structure of the detected RLKs

Shown are the PFAM domains identified by InterPro and the signal peptide (SP) and transmembrane domains (TM), in addition to four types of ectodomains and the protein kinase domain. RLK families (second column) were assigned based on the metaRLK database (Zhang et al., 2026) and the homology to Arabidopsis LRR-RLKs. The RLKs are grouped based on their relative accumulation in *rd21* mutants (last column).

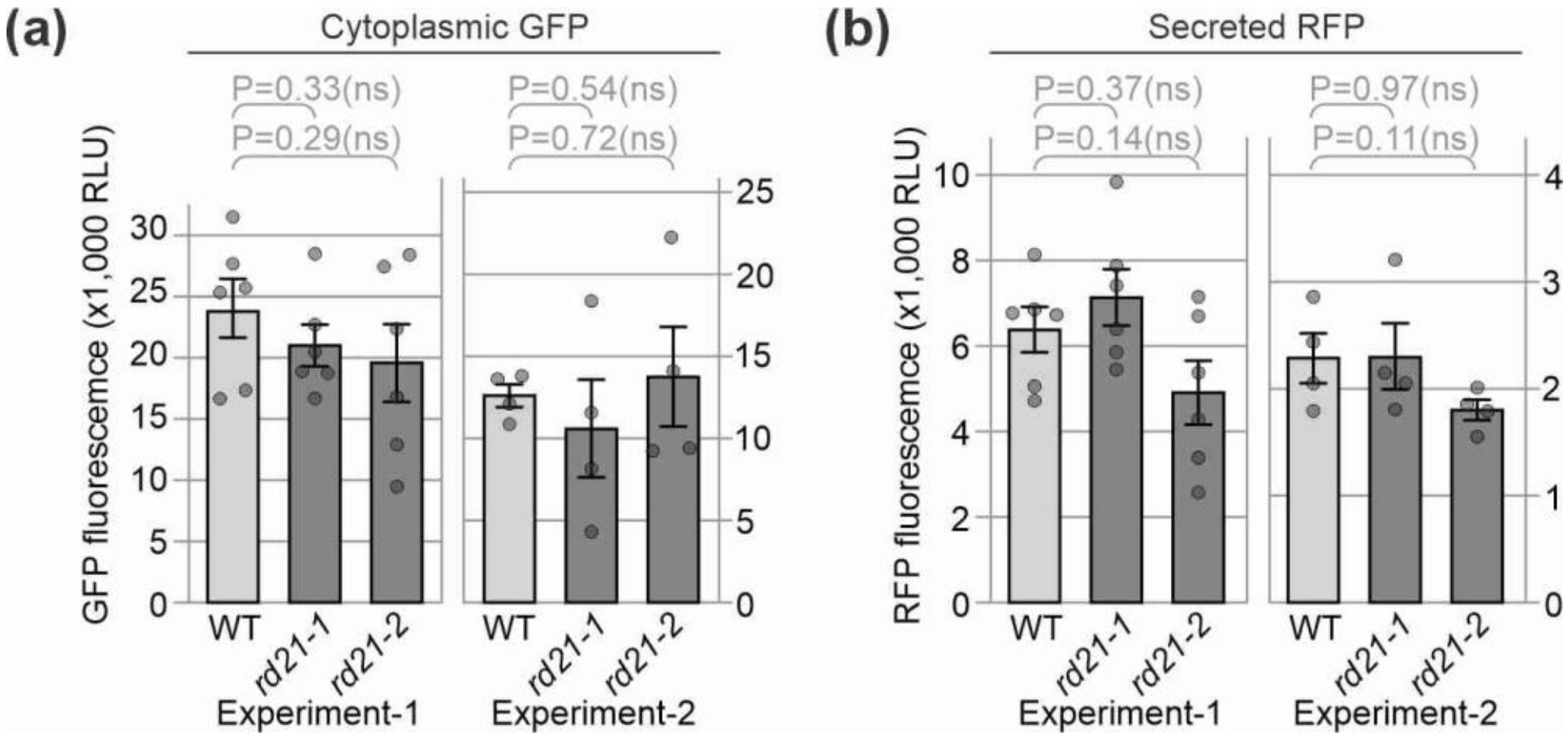

Fig. S8 No differential cytoplasmic GFP or secreted RFP accumulation in *rd21* mutants.

(a) Quantified GFP fluorescence upon transient expression of cytoplasmic GFP (cGFP) is unaltered in *rd21* plants. **(b)** Quantified RFP fluorescence upon transient expression of secreted RFP (sRFP) is unaltered in *rd21* plants. (A-B) cGFP and sRFP were transiently expressed in WT and *rd21* plants and images were taken three days later. Bars represent SE of n=6 and n=4 plants of two experiments, respectively. P-values were calculated by Student's t-test and are non-significant (ns).

Table S1 Used oligonucleotides

**Name**	**Sequence (5'-3')**
C161A-F	CTGTGGGAGTGCTTGGGCATTCTCTGCAATTGC
C161A-R	GCCCAAGCACTCCCACAGCTTCCTTGATCC
sgRNA1	GCAGTGAACTCGATAGTCAC
sgRNA2	GGTGTGGTAGCTGTTGGATA
NbL08g19980-F	GACTCGACGGGCCTTCTAAA
NbL08g19980-R	AGAAAGAATGGCAAGCAACATGC
NbL17g14770-F	GTGGGAGATGCGACCAAAATCG
NbL17g14770-R	AGATGGAGGAGAACGGCCAG
NbL04g16800-F	GTTTGCCGGATGCCGTTGACTGG
NbL04g16800-R	AGGTGGCTTGACTGGTGAAGG
GFP-F	GGTGGCATGGTGAGCAAGGG
GFP-R	ACCTCCTGCACATCAACAAA
FER2-F	TTTGTTGATGTGCAGGAGGTCAAAATTCTGCAACTGGTGA
FER2-R	CCCTTGCTCACCATGCCACCGCGTCCTTTTGGATTCATGA
BAM1-F	TTTGTTGATGTGCAGGAGGTAGACAACCCCGTATACCTGA
BAM1-R	CCCTTGCTCACCATGCCACCGATGCTGAGTAGGTCAGGTG
NbeIF4A-14_F	TGACAGAGCTGCTTGGGACT
NbeIF4A-14_R	TGGCTTCTCAAAACCATAAGCATAGAT
NbHAK1_F	CCCTAAAGTGTTGCCAGGACAA
NbHAK1_R	TTGTCCAAAGGATCAGCATCAGTATA
NbBRI1_F	CATTAAGCATCAACCTTGCCGC
NbBRI1_R	CCACCAGAGCCGATAAGACTATC
NbFER1_F	AGTTTCTTGCTGATTCTTTAGTGAC
NbFER1_R	CGACAACAGCCAACCACAGG
NbBAM1_F	TGTTGCTGATTTTGGACTTGCT
NbBAM1_R	GCGTGTAAGCATATTCTGGAGC

Table S2 Used plasmids

Name	Description	Reference
pJK001c	Empty vector	Paulus et al., 2020
P19	Binary 35S::P19	Van der Hoorn et al., 2003
pPB34	Binary 35S::*Nb*RD21	Sueldo et al., 2024
pAG11	Binary 35S::C161A	This work
pPJ064	Binary RD21 genome editing plasmid	This work
pMS21	Binary 35S::FER2-GFP	This work
pMS24	Binary 35S::BAM1-GFP	This work
pJK227	Binary 35S::Cf-4-GFP	Kourelis et al., 2020
pJK309	Binary Cas9 vector	This work
pID41	Binary Cytoplasmic 35S::GFP (no p19)	Dodds et al., 2025
pID42	Binary Secreted 35S::RFP (no p19)	Dodds et al., 2025
